# Stranger Rape or Impromptu Consensual Sex? Investigating Mock Juror Decision‐Making in a Genuine Contested Rape Trial

**DOI:** 10.1002/bsl.70032

**Published:** 2025-12-10

**Authors:** Dominic Willmott, Rosie Woodhams

**Affiliations:** ^1^ Department of Criminology, Sociology and Social Policy Loughborough University Loughborough UK; ^2^ Faculty of Psychology SWPS University Wroclaw Poland

**Keywords:** juror bias, jury decision‐making, rape myths, sexual violence, stranger rape

## Abstract

The aim of this study was to better understand juror decision‐making in a less typical rape trial scenario where even prior acquaintance is disputed. Adopting an improved mock trial paradigm including a video‐recorded recreation of a genuine rape allegation and jury‐group deliberation, 156 jury‐eligible participants took part in 1 of 13 identical 12‐person mock trials. Pre‐trial, a psychosocial questionnaire was conducted and post‐trial, juries deliberated attempting to reach a unanimous verdict. Regression analyses revealed that male jurors, those with greater belief in rape myths and lower scores in interpersonal manipulation were most likely to return *not guilty* verdicts pre‐deliberation. Post‐deliberation, increased self‐esteem and rape myth acceptance scores were associated with *not guilty* verdict selections. Female and Caucasian jurors were most likely to change their decision following group‐deliberation. This research has important implications for understanding the role that juror biases can have on rape trial outcomes with jury reform initiatives discussed.

## 1 Introduction

### 1.1 Rape Prevalence and Attrition

Sexual violence is a highly prevalent social issue, with the 2022 Crime Survey for England and Wales estimating 1 in 4 women and 1 in 18 men experience rape or sexual assault (ONS 2023). Of these instances, the majority are never reported to the police (K. Daly and Bouhours 2010; Stewart et al. 2024; Wolitzky‐Taylor et al. 2010), producing what has long been termed the ‘dark figure’ of sexual offending (Biderman and Reiss 1967, 1). Those who do report face considerable barriers to justice, with high rates of attrition through cases being dropped by the police (George and Ferguson 2021; Haynes and Kebbell 2025; Zvi 2022), discontinued by prosecutors (Willmott et al. 2021) or where victim‐survivors withdrawal from the process due to their treatment following a report (Brown et al. 2007; Sinclair 2022). Indeed, strong public opinion and general doubt surrounding the veracity of rape allegations (see Smith et al. 2022; Weare et al. 2025) display why complainants often feel a retraction is the least bad option.

Criminal justice responses to rape have faced scrutiny for consistently low conviction rates despite increasing reports (Dalton et al. 2022; George and Ferguson 2021), leading critics to claim that rape has effectively been decriminalised in the UK (EVAWC 2020). Exacerbated by lengthy timeframes (Evennett 2025), only 2.7% of rapes recorded by police between October 2023 and September 2024 resulted in a charge within that same year, let alone conviction (Home Office 2025). Although the overall conviction percentage showed small improvements from 2016 to 2022 (CPS 2017, 2022), the Crown Prosecution Service (CPS) has been accused of selectively charging very few rape complaints in order to skew their overall conviction ‘success’ rate (see EVAWC 2020; George and Ferguson 2021). Available statistics appear to support this claim, with a 37.4% drop prosecutions from 2015–2016 to 2019–2020 (CPS 2019, 2020). Regardless, the conviction rate remains considerably lower than for other offences, with the most recent CPS data showing just 58% of rape‐flagged offences resulted in a conviction at trial, compared to 83% of offences overall (CPS 2024).

### 1.2 Rape Myths and Juror Decisions: A Methodological Feedback Loop

One widely attested barrier to improving rape convictions is the prevalence of rape myths at all stages of the criminal justice process (Dinos et al. 2015; Ferreira et al. 2025; Horvath and Davies 2025; Grzyb et al. 2025; Lovell et al. 2025; Parratt and Pina 2017; Woodhams 2023). Rape myths (RMs) are ‘prejudicial, stereotyped, or false beliefs about rape, rape victims, and rapists’ (Burt 1980, 217) which act as a means of justifying or minimising sexual violence (Lonsway and Fitzgerald 1994). These beliefs are not necessarily outright false in all instances; however, their generalisation to all instances of rape is what means that they are considered myths (Leverick 2020; Snow and Longpré 2025). For example, a common myth is that rape is largely perpetrated by strangers yet only in around 15% of rapes against women in England and Wales (E&W) is the alleged perpetrator a stranger to the complainant (ONS 2021). In comparison, 45% are perpetrated by a current or former partner (ONS 2021), with these instances facing even lower chances of conviction (Hester and Lilley 2017; Willmott and Hudspith 2024).

Numerous analyses have explored the impact of RMs on jury decision‐making, finding a consistent association between greater belief or endorsement of various rape mythology pretrial and jurors perception that the defendant's account is more credible that the complainant's (Klement et al. 2018; Willmott 2018) alongside a preference for not guilty verdicts (Dinos et al. 2015; Lilley et al. 2023; Willmott et al. 2018; Mojtahedi et al. 2024; Stevens et al. 2024). Although it has been argued that jury group deliberations *may* mitigate any preconceived biases among jurors (Kapardis 2014; Thomas 2008), successive studies conclude that jurors pre‐trial rape myth beliefs appear to have a consistently prejudicial influence on rape trial verdict outcomes irrespective of group deliberation (Richardson et al. 2025; Willmott, Boduszek, et al. 2026). Therefore, there have been increasing calls for reform to the jury system, from greater use of judicial directions to address myths (see Weare et al. 2025) to more radical reforms including that jurors be vetted to assess rape myth attitudes before trial (see Willmott 2018). Indeed, in Scotland juries were very nearly removed altogether for rape cases, with a pilot initiative halted at the last minute that would have seen rape trials presided over exclusively by a lone trial judge, despite concerns about the efficacy of such a ‘solution’ (see Curley and Neuhaus 2024; Gu 2024).

Conversely, in one of the only studies granted access to real jurors through commission by the UK Ministry of Justice, it is argued that predisposed attitudes and beliefs towards rape were much less influential than mock jury research suggests (Thomas 2020). In her 2020 study, Thomas presented 771 discharged jurors with statements about rape and rape victims, asking them to declare the extent to which they agree on a three‐point scale (agree, disagree, unsure). For example, ‘a rape probably didn't happen if the victim has no bruises or marks’ (see Thomas 2020 for all statements used). Despite varied levels of endorsement between statements, reportedly, overall findings indicated much lower rape myth endorsement than suggested by previous research. This led Thomas leading to conclude that ‘previous claims of widespread “juror bias” in sexual offences cases are not valid’ (Thomas 2020, 24), asserting the mock jury research is not a reliable estimator of genuine jury decision making.

Although some academics appear to support these assertions (see Waiton 2023) and share Thomas' critiques of certain mock jury research (Ross 2023, 2024), her work has faced extensive scrutiny for both its methodological validity and the legitimacy of the conclusions drawn from its findings (see Chalmers et al. 2021; E. Daly et al. 2023; Willmott and Hudspith 2024). While gaining access to real jurors is a clear strength of the research and a rarity due to strict legal restrictions that govern access to genuine trial jurors (Willmott, Curley, et al. 2026), asking jurors to retrospectively self‐report their agreement with a small number of rape myth statements post‐trial is entirely different from assessing rape myth beliefs using a comprehensive psychometrically validated rape myth acceptance scale (e.g., Persson et al. 2026), within the context of an applied rape case where jurors are questioned following a highly realistic live rape trial re‐enactment (e.g., Weare et al. 2025; Willmott 2018). Furthermore, Thomas research, like much mock jury research, fails to scrutinise what occurs during jury deliberations—a significant oversight given other highly realistic (mock) jury research indicates that rape myths remain front and centre within jury group deliberations (Ellison and Munro 2010; Richardson et al. 2025). Thus, despite her conclusions, Thomas (2020) study findings do not discern, or in fact directly test, whether rape myth beliefs have any influence upon individual juror decisions or collective jury verdicts.

### 1.3 Juror Traits, Attitudes and Decisions

Although mock jury research often includes measures of demographic and personality traits, they are generally considered to be weak predictors of verdict outcomes in criminal cases (Lieberman and Krauss 2016) when compared to crime‐specific attitudes (Willmott, Boduszek, et al. 2026). Despite this, empirical attempts to determine whether any association exists between such psychosocial variables and juror verdicts persist, especially within specific case types. Regarding gender, research typically indicates that female jurors are more conviction prone in sexual offence trials when compared to their male counterparts (Kovera et al. 1999; Willmott 2018), especially when alleged offences are perpetrated against children (Bottoms et al. 2014; McCoy and Gray 2007; Pettalia et al. 2017). However, with some research failing to find such an association, further research is needed to test the existence of such a relationship in varied sexual offence case types (for a recent review on see Millar et al. 2025).

When examining any possible association between juror ethnicity and subsequent decision making, empirical literature is even more mixed. Though often identified as important when considered alongside the ethnicity of the defendant, with jurors showing greater leniency for those from the ethnic group as them, to a point (Bottoms et al. 2004; N. J. King 1993; Perez et al. 1993), when controlling for the influence of defendant and complainant race, other studies suggest this relationship may be less clear cut. Moreover, while some studies found no effect of ethnicity on juror decision making in rape trials (Curley et al. In Review), others found juror race to be important; with Black African‐Caribbean and South Asian heritage jurors less likely to convict rape defendants than their Caucasian counterparts (Lilley et al. 2023).

Also somewhat unclear, is the role of prior sexual victimisation experiences upon verdict preferences. Jones et al. (2020) observed that in child sexual abuse trials, jurors with relevant personal experience (either first‐hand experience or someone close to them) displayed heightened empathy towards complainants and were more likely to assign guilt to defendants overall. Some recent research appears to corroborate this association, finding jurors with sexual victimisation experiences were more likely to return guilty verdicts in rape trials (Lilley et al. 2023; Lathan et al. 2025) whereas other research found no evidence of any such effect (Grandgenett et al. 2022; Headd and Willmott 2025; Willmott 2018). Since mock juror research participation is typically dominated by student samples, links between educational attainment and verdict selections are often drawn from comparison between juror verdicts from student and community samples. This has yielded inconsistent results with some studies finding student samples to be more lenient (McCabe et al. 2009), others more punitive (Martín et al. 2007; Warling and Peterson‐Badali 2003), and some reporting no differences at all (Bornstein et al. 2017) warranting further exploration.

Finally, although scant research has explored the impact of personality traits including egocentrism and interpersonal manipulation on juror decisions making, the psychopathology of jurors has been explored in a small number of studies. For example, in one study, mock jurors with affective trait empathy deficits were found to be those most likely to convict acquaintance rape defendants, attributed to their lack of consideration of the impact that a rape conviction may have on the defendants life (Lilley et al. 2023), found to be a common barrier to conviction in studies which analyse jury deliberations (e.g., Richardson et al. 2025; Weare et al. 2025). However, further research is needed to examine the role of any such traits in jurors rape trial decision making given that the lack of evidence to support or refute such an effect. Similarly underexplored is the influence of juror self‐esteem on trial decision making. Although rarely explored in mock jury research, decreased juror self‐esteem was associated with increased belief in a rape defendant's testimony in one study (Willmott 2018). In this study the authors also found an association between juror self‐esteem and verdict decision confidence, which they suggest requires further exploration, speculating that juror self‐esteem levels may mean that certain jurors are more susceptible to group influence during deliberation. Clearly further research is warranted to explore the role of self‐esteem in juror decision making. Parenthood is also variable yet to be considered in mock jury research. While being a parent to a child has been associated with more punitive attitudes to convicted sex offenders (L. L. King and Roberts 2017), whether this translates to jurors who are parents being more guilty verdict prone in contested rape cases is yet to be established in empirical research.

### 1.4 Study Aims and Rationale

In light of the lack of consensus surrounding which variables may be associated with juror decision‐making in rape trials (and to what extent), in the current study we aim to advance existing knowledge by exploring the impact of psychosocial traits and predisposed attitudes on juror verdict sections both before and after jury group deliberation. Here we test the possible influence of such variables within the context of a genuine (though less typical) rape scenario, where even prior acquaintance is contested between the two concerned parties. In consideration of vast criticism around the methodological validity of mock juror research (Bornstein 1999; Ross 2024; Willmott, Boduszek, et al. 2026; Willmott, Curley, et al. 2026), the present study also utilises an improved mock trial paradigm with a videotaped mock trial developed from a genuine case, and with input from practicing legal professionals. By adopting a mock trial reconstruction that includes jury group deliberation and a real courtroom environment, among other methodological enhancements detailed at length below, this study aims to enhance our understanding of jury decision making within rape trials within the context of a mock trial paradigm that exhibits increased ecological validity to; *examine the relationship between pre‐trial juror rape attitudes and varied psychosocial traits upon verdict selections made pre and post‐deliberation*.

## 2 Methods

### 2.1 Sample and Sampling Procedure

A self‐selecting sample of 156 participants were opportunistically recruited through an advertisement placed throughout a small British town and university campus. Prospective participants emailed the lead researcher to register their interest and were subsequently sent a weblink to an event management website describing the nature of the study, the task that mock jurors were asked to undertake, and a range of mock trial dates scheduled over an 8‐week period (15 advertised in total). From the dates listed participants were asked to select one date/time and encouraged to do so in isolation (i.e., to avoid booking slots that friends or known peers were also attending). Before signing up to any scheduled mock trial, jury‐eligibility questions were asked in accordance with current criteria in E&W. The final sample of mock jurors was confirmed to be; (1) aged 18–75 years old, (2) had no serious criminal offence history, (3) were not suffering from severe mental health illness and otherwise (4) eligible to vote in local and government elections (i.e., they had lived in the UK for at least 5 years since their 13th birthday). Participants who did not meet these jury‐eligibility criteria based upon responses provided were unable to book a place onto any of the 15 advertised trial dates. Two of the scheduled mock trials had to be cancelled on the day as they did not recruit the 12‐jurors required to proceed however the remaining 13 mock trials went ahead as planned after 12‐jurors registered for each. A reserve list of prospective mock jurors who expressed an interest in taking part after all spaces had been filled was used to replace jurors who dropped out before the day of each trial. However, regular reminders were sent to the participants who registered for each trial to reduce late dropouts. The final sample thereby included 156 mock jurors across 13 separate 12‐person jury panels.

Participants were aged 18 to 55 (*M =* 23.48, SD = 6.70) and were largely female (62.8%) and Caucasian (62.2%). A small number of participants reported being a parent to at least one child (10.3%). Regarding educational attainment, most participants (73.1%) reported their highest qualification at the time of the study as being less than a university bachelor's degree and this included university students currently studying for an undergraduate bachelor's degree qualification that had not yet been completed. A quarter of participants (26.9%) reported their highest qualification being a bachelor's degree or above (e.g., master's or doctorate degrees). Mock jurors were also asked about past victimisation experiences and a significant minority reported having been a victim of crime at least once (26.9%), with a small proportion reporting being the victim of a serious crime (4.5%) and a sexual crime such as rape (2.6%). More than a quarter of the sample (26.9%) reported knowing a friend or family member that had experienced a sexual crime such as rape.

### 2.2 Measures and Materials

#### 2.2.1 Acceptance of Modern Myths About Sexual Aggression (AMMSA)

The AMMSA (Gerger et al. 2007) is a self‐report unidimensional 30‐item scale developed to measure modern rape myth acceptance and attitudes held towards sexual aggression in diverse populations (e.g., ‘*Alcohol is often the culprit when a man rapes a woman*’). Responses are measured on a seven‐point Likert scale (1 = Completely Disagree to 7 = Completely Agree) with total scores ranging from 30 to 210. Higher scores indicate greater acceptance of modern myths surrounding sexual aggression (Cronbach's alpha = 0.92).

#### 2.2.2 Rosenberg Self‐Esteem Scale (RSES)

RSES (Rosenberg 1965) is a 10‐item unidimensional self‐report measure of self‐esteem, designed to capture positive or negative feelings held in relation to oneself (e.g., ‘*On the whole, I am satisfied with myself*’*,* ‘*At times, I think I am no good at all*’). Responses are measured on a four‐point Likert scale (1 = Strongly Disagree to 4 = Strongly Agree) with possible scores ranging between 10 and 40. Higher scores reflect more positive evaluations of the self, thus indicating greater self‐esteem (Cronbach's alpha = 0.87).

#### 2.2.3 Psychopathic Personality Traits Scale (PPTS)

The PPTS (Boduszek et al. 2016) is a 20‐item self‐report measure of psychopathic personality traits developed as a non‐diagnostic research measure to be used among offending and non‐offending populations. The scale measures psychopathic personality traits through four distinct yet conceptually related constructs: Affective Responsiveness (AR; 5 items, *α* = 0.86), that captures characteristics of low affective empathy and emotional shallowness (e.g., ‘*What other people feel doesn't concern me*’); Cognitive Responsiveness (CR; 5 items, *α* = 0.76), that reflects a person's ability to understand the emotional state of others and emotionally engage with them at a cognitive level (e.g., ‘*I am good at predicting how someone will feel*’); Interpersonal Manipulation (IPM; 5 items, *α* = 0.84), which measures manipulative tendencies including superficial charm, deceitfulness and grandiosity (e.g., ‘*I sometimes provoke people on purpose to see their reaction*’); and Egocentricity (EGO; 5 items, *α* = 0.69), which captures a person's tendency to focus on their own attitudes, beliefs and interests (e.g., ‘*I believe in the motto: I'll scratch your back, if you scratch mine*’). Responses are measured on a 5‐point Likert scale (1 = ‘strongly disagree’ to 5 = ‘strongly agree’). For AR and CR factors, increased scores indicate a lack of each type of empathy, whereas higher scores on EGO and IPM factors indicate increased trait tendencies.

#### 2.2.4 Demographic, Victimisation and Verdict Decision Information

A self‐report demographic questionnaire was devised to collect information regarding participants' age, gender, ethnicity, current highest educational qualification and victimisation experiences. Age was recorded as interval data, while all other demographic variables were recorded as nominal data. Ethnicity responses were recoded as Caucasian and Black Asian Minority Ethnic origin (typically abbreviated to BAME in the UK). Merging non‐Caucasian participants into a BAME category is not our preferred method of examining ethnicity differences between jurors however, due to a low cell count across a diverse range of ethnic identities included in the sample (e,g, Black Caribbean and/or Black African heritage, South Asian heritage including Indian, Pakistani, Bangladeshi, Chinese, and bi‐racial or Arabic heritage), this was necessary to provide some (albeit limited) comparison. Four questions related to participant exposure to victimisation (e.g., *experiences of crime, serious crime, sexual crime such as rape or knowing friends or family that had*) were also asked and measured dichotomously (yes/no). Verdict decisions to the question ‘*How do you find the defendant, on the allegation that he raped the complainant?*’ were also binary coded (guilty/not guilty) according with genuine verdict categories used at trial in E&W.

#### 2.2.5 Mock Trial Materials

Participants were presented with case information via a videorecorded mock trial reconstruction, part of which was filmed within a real Crown courtroom in the North of England. The trial depicted was a genuine contested rape allegation. Using a trial transcript the mock trial was presented as follows; first, jurors were presented with the undisputed facts in the case outlining that a sexual interaction occurred between one female complainant and one male defendant who are alleged to have met for the first time just a few minutes before the alleged rape occurred. The complainant argues that an unknown male followed her into a dark secluded area (a place where she had momentarily gone to urinate) after leaving a busy nightclub and proceeded to rape her. In contrast, the defendant argues that following a chance meeting with the complainant while they were exiting the nightclub, they arranged an impromptu consensual sexual encounter in the secluded area where the rape is said to have occurred. After setting out the agreed facts and key differences in each of their accounts, a summary of the prosecution case (key arguments) was presented to jurors, which included an audio recording of the complainant's testimony (recreated by an actor), followed by a summary of examination‐in‐chief (by the prosecution) and cross‐examination of the witness (by the defence). The main prosecution argument being that the defendant was lying about the pre‐arranged meet for consensual sex and had instead simply taken advantage of a lone intoxicated women in a secluded location and decided to rape her. Next, the defendant's account, again presented as an audio recording by an actor, followed by key arguments advanced by the defence and a summary of examination‐in‐chief, cross‐examination and re‐examination. The main defence argument was that the defendant had engaged in what he believed to be consensual sex, arguing that it was the complainant who solicited him to have consensual sex, a request that he had simply enthusiastically complied with. The defence argue that the allegation of rape emerged because after sexual intercourse occurred, the defendant had promptly left the complainant after he had got what he wanted. Next, mock jurors were presented with a summary of the medical evidence in the case that outlined while there was some evidence of injuries found to the complainant's genitals, it could not be determined whether these injuries were the result of consensual or non‐consensual sexual activity. Finally, mock jurors were presented with the judge's summary of the case and final instructions that jurors were asked to follow, depicted by an experienced criminal barrister who acted out the role of the judge. The judge's instructions were crafted in close consultation with a genuine rape ticketed crown court judge from England and using judicial guidance documents used by English judges when developing jury directions in real trials (i.e., the Crown Court Compendium). An expert panel comprising of Rape and Serious Sexual Assault (RASSO) police detectives, prosecution and defence lawyers, and a serving English trial judge were consulted throughout the mock trial study such that the trial reconstructions would be closely aligned with UK legal practice and law of evidence. The final condensed video tapped trial was 24 min long.

### 2.3 Study Procedure

An experimental mock trial paradigm was employed where participants were recruited to take part in 1 of 13 identical mock trial reconstructions held within a mooting courtroom on a university campus. On arrival mock jurors were welcomed and seated in the courtroom. Once all 12 mock jurors were in attendance, participants were provided with a paper and pencil questionnaire booklet containing an information sheet, consent form, and a psychosocial survey including questions and scales described in the measures section above. Upon providing their informed consent, mock jurors were asked to complete the pre‐trial questions. This process took participants between 20 and 30 min. After collecting in the completed pre‐trial questionnaires, jurors were informed that the trial was about to begin and given the opportunity to ask questions. They were also informed that if they would like to write notes while observing the trial video that they could. After observing the trial and prior to discussing the case as a group, individual jurors were asked to indicate their pre‐deliberation verdict decision (guilty or not guilty). Next, participants were presented with deliberation instructions and encouraged to appoint a jury foreperson to mediate discussions and facilitate their efforts to reach a unanimous verdict decision. Participants were then asked to leave the jury box area of the mooting court and directed to a separate deliberation room as a group. At this stage, all researchers left the room to allow mock jurors to deliberate in private asking that the foreperson call the experimenter back into the room once a collective verdict had been decided. After a period of 30 min juries that had not reached a collective verdict were informed that a majority decision (10/2) could now be accepted (known as a Watson direction) and where a majority decision could not be reached, the foreperson would have to declare a hung jury. After all deliberations concluded and jury panels delivered their group verdicts, participants were asked once again to indicate their final individual post‐deliberation verdict decisions. Here, jurors were reminded that their final individual verdict decision may or may not reflect that of the collective jury group decision and that this would not be shared with their fellow jurors. As such, they were encouraged to be honest in sharing the verdict that they personally felt to be accurate at this final stage. Finally, mock jurors were then informed that the trial was over, were debriefed and thanked for taking part. Each experiment lasted between 100 and 175 min from arrival to debriefing. Participants did not receive any reward or compensation for taking part.

## 3 Results

Descriptive statistics for all scale data and frequency distributions for nominal data are presented in Tables 1 and 2, respectively. Individual verdict decision data display that most jurors opted for a *not guilty* verdict decision before discussing the case with their fellow jury panel members (64.1%), with individual jurors not guilty verdict selections increasing further following group deliberation (72.4%). However, a substantial minority of mock jurors arrived at a guilty verdict both before (35.9%) and after (27.6%) group deliberation indicating some variability in the verdict decisions that individual jurors made. Collective jury‐group verdict decisions across the 13 separate panels concluded with nine unanimous *not guilty* verdicts, two unanimous *guilty* verdicts and two jury panels ending in a hung verdict after declaring that they were unable to reach a majority consensus of at least 10–2.

**TABLE 1:** Descriptive statistics for age, AMMSA, and PPTS subscales (*N* = 156).

Variables	*M*	SD	Observed min	Observed max
Age	23.48	6.70	18.00	55.00
AMMSA	97.34	27.12	39.00	155.00
AR	10.31	3.55	5.00	22.00
CR	10.44	3.03	5.00	18.00
IPM	13.15	3.76	5.00	24.00
EGO	13.04	3.10	7.00	20.00
RSES	20.28	4.19	10.00	37.00

Abbreviations: AMMSA, Acceptance of Modern Myths about Sexual Aggression total score; AR, Affective responsiveness total score; CR, Cognitive Responsiveness total score; ECO, Egocentricity total score; IMP, Interpersonal Manipulation total score; RSES, Rosenburg Self Esteem Scale total score.

**TABLE 2:** Frequency distribution of categorical participant data (*N* = 156).

Variables	Sample	Guilty verdicts (pre‐deliberation)	Guilty verdicts (post‐deliberation)
Gender			
Female	98 (62.8%)	43 (43.9%)	31 (31.6%)
Male	58 (37.2%)	13 (22.4%)	12 (20.7%)
Ethnicity			
Caucasian	97 (62.2%)	37 (38.1%)	28 (28.9%)
BAME	59 (37.8%)	19 (32.2%)	15 (25.4%)
Highest qualification			
Less than bachelors	114 (73.1%)	43 (37.7%)	37 (32.5%)
Bachelor's and above	42 (26.9%)	13 (31.0%)	6 (14.3%)
Parent to child			
Yes	16 (10.3%)	6 (3.8%)	6 (3.8%)
No	140 (89.7%)	50 (32.1%)	37 (23.7%)
Victim of crime			
Yes	42 (26.9%)	17 (40.5%)	16 (38.1%)
No	114 (73.1%)	39 (34.2%)	27 (23.7%)
Victim serious crime			
Yes	7 (4.5%)	6 (85.7%)	4 (57.1%)
No	149 (95.5%)	50 (33.6%)	39 (26.2%)
Victim sexual crime			
Yes	4 (2.6%)	4 (100.0%)	2 (50.0%)
No	152 (97.4%)	52 (34.2%)	41 (27.0%)
Friends/fam victim			
Yes	42 (26.9%)	18 (42.9%)	14 (33.3%)
No	114 (73.1%)	38 (33.3%)	29 (25.4%)
Total	156 (100%)	56 (35.9%)	43 (27.6%)

Abbreviations: BAME, Black‐Asian Minority Ethnic origin; Friends/fam victim, Do you have any friends or family members that have experienced a sexual office such as rape?.

### 3.1 Effects of Deliberation

The data indicated that 12.2% of the sample (*n* = 19) changed their verdict after deliberation, with three participants changing from not‐guilty to guilty verdicts, with a larger number of jurors (*n* = 16) changing their pre‐deliberation guilty verdicts to not‐guilty. The impact of deliberation was thereby seemingly most evident among those jurors whose pre‐deliberation belief in guilt became less certain following group discussions with their fellow jurors, leading them to revise their individual verdict selection post‐trial. A closer look at the 19 jurors who did change their mind following deliberations indicates that those most likely to concede to the influence of other jurors were; female (73.7%), Caucasian (68.4%) and reported their highest qualification as below a university bachelor's degree (63.2%). No significant differences were found in age, rape myth acceptance or self‐esteem scores. Importantly however, while a minority of the sample of jurors changed their individual verdict decision following group deliberation (10.3%), the vast majority (89.7%) did not.

### 3.2 Predicting Verdict Selections

Two binary logistic regression models were tested to determine the association between age, gender, ethnicity, educational attainment, parental status, previous victimisation experience, personality traits (AR, CR, IPM, EGO, RSES) and rape myth acceptance (AMMSA) on verdict decisions both pre‐ and post‐deliberation.

The model for pre‐deliberation verdicts was statistically significant, *χ* ^2^ ((df *=* 12, *N =* 156) = 25.43, *p <* 0.05, Cox & Snell *R* ^2^ = 0.150, Nagelkerke *R* ^2^ = 0.206), indicating that the model could distinguish participants who returned guilty verdicts from those that returned not guilty verdicts. The model as a whole correctly identified 64% of pre‐deliberation responses. As displayed in Table 3, AMMSA (OR = 0.97, *p* < 0.01), IPM (OR = 1.16, *p* < 0.05) and gender (OR = 0.33, *p* < 0.05) made a statistically significant contribution to the model. These findings indicate that mock jurors who displayed decreased rape myth acceptance scores, heightened interpersonal manipulation scores and are female, were those most likely to return guilty verdicts pre‐deliberation. Alternatively put, jurors who scored high in their endorsement of rape myths, scored low in interpersonal manipulation, and were male, were significantly less likely to return a guilty verdict.

**TABLE 3:** Binary logistic regression model for verdict decisions pre‐deliberation (*N* = 156).

Variables	*B*	SE	OR (95% CI)
AMMSA	−0.03	0.01	0.97** (0.96/.99)
AR	0.05	0.07	1.06 (0.92/1.21)
CR	0.01	0.08	1.01 (0.87/1.18)
IPM	0.15	0.07	1.16* (1.01/1.32)
EGO	−0.08	0.08	0.93 (0.79/1.09)
Age	−0.04	0.04	0.97 (0.89/1.05)
RSES	−0.06	0.05	0.95 (0.86/1.04)
Gender (male)	−1.10	0.47	0.33* (0.13/.84)
Highest qual (bachelors+)	−0.02	0.52	0.98 (0.35/2.73)
Ethnicity (Caucasian)	−0.35	0.45	0.71 (0.30/1.69)
Parent (yes)	−0.30	0.93	1.35 (0.22/8.38)
Victim of crime (yes)	0.57	0.47	1.76 (0.70/4.45)

Abbreviations: 95% CI, Confidence Interval; AMMSA, Acceptance of Modern Myths about Sexual Aggression total score; AR, Affective responsiveness total score; CR, Cognitive Responsiveness total score; ECO, Egocentricity total score; IMP, Interpersonal Manipulation total score; OR, Odds Ratio; RSES, Rosenburg Self Esteem Scale total score; SE, Standard Error.

* *p* < 0.05.

** *p* < 0.01.

***p < 0.001.

A second test of the complete model was carried out to examine the correlates of individual juror verdict decisions given post‐deliberation. The model was statistically significant, *χ* ^2^ ((df *=* 12, *N =* 156) = 30.64, *p <* 0.01, Cox & Snell *R* ^2^ = 0.18, Nagelkerke *R* ^2^ = 0.29), indicating that the model could distinguish between individuals who returned guilty verdicts and those who returned not‐guilty verdicts. The model correctly identified 77.6% of responses.

Displayed in Table 4, two variables made a statistically significant contribution to the model including AMMSA scores (OR = 0.97, *p* < 0.01) and self‐esteem scores (OR = 0.84, *p* < 0.01). Here, results identify that participants who exhibited lower scores in self‐esteem and rape myth acceptance were those most likely to return guilty verdicts following jury deliberation. Alternatively put, jurors who exhibited higher self‐esteem and greater belief in rape myths were significantly less likely to return a guilty verdict in this case.

**TABLE 4:** Logistic regression model for verdict decisions post‐deliberation (*N* = 156).

Variables	*B*	SE	OR (95% CI)
AMMSA	−0.03	0.01	0.97** (0.96/.99)
AR	0.04	0.08	0.96 (0.82/1.13)
CR	0.04	0.09	1.05 (0.88/1.24)
IPM	0.09	0.07	1.09 (0.96/1.25)
EGO	0.02	0.09	1.02 (0.86/1.21)
Age	−0.08	0.05	0.92 (0.84/1.01)
RSES	−0.17	0.07	0.84** (0.74/.96)
Gender (male)	−0.48	0.52	0.62 (0.23/1.71)
Highest qual (bachelors+)	−0.97	0.65	0.38 (0.11/1.34)
Ethnicity (Caucasian)	−0.29	0.48	0.75 (0.29/1.90)
Parent (yes)	1.34	0.99	3.83 (0.55/26.82)
Victim of crime (yes)	0.98	0.51	2.66 (0.99/7.17)

Abbreviations: 95% CI, Confidence Interval; AMMSA, Acceptance of Modern Myths about Sexual Aggression total score; AR, Affective responsiveness total score; CR, Cognitive Responsiveness total score; ECO, Egocentricity total score; IMP, Interpersonal Manipulation total score; OR, Odds Ratio; RSES, Rosenburg Self Esteem Scale total score; SE, Standard Error.

* *p* < 0.05.

** *p* < 0.01.

***p < 0.001.

### 3.3 Comparing Rape Myths Between Juror Verdicts

Independent samples *t*‐tests were conducted to compare AMMSA scores between verdict selections both pre‐ and post‐deliberation with results supporting those reported in the logistic regression results above. Pre‐deliberation findings display that AMMSA scores differed significantly (*t* = −3.111, df = 154, *p* = 0.001, *d* = 0.52) between those who returned guilty (*M* = 88.55. SD = 25.79) and not guilty (*M* = 102.26. SD = 26.73) verdicts. A consistent finding was also observed post‐deliberation in that AMMSA scores differed significantly (*t* = −2.433, df = 154, *p* = 0.001, *d* = 0.50) between those who returned guilty (*M* = 88.91. SD = 23.93) and not guilty (*M* = 100.55. SD = 27.67) verdicts.

## 4 Discussion

The current study sought to shed light on the variables affecting jury decision‐making in a less typical case where even prior acquaintance was contested. Overall, the findings corroborated much of the previous literature, and contributing unique insights into the impact of lesser‐explored variables, within the context of methodological enhanced and ecological valid mock trial settings. Moreover, consistent with previous research that suggests jurors tend to favour not guilty outcomes in contested rape cases (Ellison and Munro 2013; Willmott et al. 2018), most jurors delivered not guilty verdicts. This was consistent across both pre‐ and post‐deliberation verdict points with a high degree of consistency. This means that, like many previous rape trial studies (e.g., Headd and Willmott 2025; Willmott, Boduszek, et al. 2026), the majority of jurors did not change their initial verdict after deliberation, countering claims that deliberation significantly mitigates any pre‐existing biases. The majority of verdicts that changed were from guilty to not guilty, a trend seen in previous similar studies (Lilley et al. 2023; Willmott et al. 2018). While this is perhaps to be expected given the burden and standard of proof to convict in E&W, there was a discernible profile to the jurors whose verdict changed. Most were female, Caucasian, and educated to below a university degree level. This is consistent with previous studies that found women were most likely to change their verdict through deliberation (Golding et al. 2007). While this could be the result of gender dynamics during deliberations, with Marder (1986) finding deliberations to be dominated by men, an alternative explanation lies in the fact that female jurors are typically found to be more conviction prone in sexual offence cases (Kovera et al. 1999; Thomas 2010; Willmott 2018). Despite this, it remains true that the male jurors almost exclusively did not shift from their initial pre‐deliberation not guilty verdict preference, though female jurors were persuaded to do so. Clearly, such a gender disparity in verdict selections and tendency to change ones decision (or not) warrants further exploration in forthcoming research, closely considering how gender dynamics and social group interactions during deliberations may explain such results.

Analysis of differences in rape myth scale scores between those who voted guilty versus not guilty demonstrated that jurors who returned not guilty verdicts scored significantly higher in rape myth beliefs than those who returned guilty verdicts. This apparent attitudinal trend underpinning verdict selection differences was further confirmed by regression analysis of verdicts both pre and post‐deliberation, evidencing that jurors who exhibited higher scores in rape myth beliefs pre‐trial were significantly more likely to return not guilty verdicts across decision points. This adds to a wealth of previous research that obtained similar results indicating an apparent prejudicial influence of rape mythology on juror decision making in rape trials (Dinos et al. 2015; Leverick 2020) and serves as further evidence that disputes Thomas' (2020) claim that rape myths are of limited concern in jury decisions.

Analysis also indicates that male jurors were significantly more likely to return not guilty verdicts than their female counterparts, again corroborating a wealth of previous research that has established that men are less believing of female sexual offence complainants (Nason et al. 2019; Root 2025) and therefore less likely to convict (Bottoms et al. 2014; Lilley et al. 2023). Given that men are more susceptible to rape myths overall and endorse varied rape mythology to a greater extent (Suarez and Gadalla 2010), this finding is not surprising and highlights those jurors most likely to benefit from targeted interventions designed to reduce the impact of rape myth bias within jury decision making. Regarding the possible impact of psychological juror traits, juror interpersonal manipulation scores were associated with verdict sections, with jurors scoring higher in manipulative tendencies found to be those more likely to return a guilty verdict prior to deliberation. In contrast to the other study findings, this contradicts the (albeit scant) existing mock jury research which reported a correlation between increased interpersonal manipulation and not guilty verdict preferences (Lilley et al. 2023). However, in the current study the role of interpersonal manipulation was not consistent post‐deliberation and so appears to have a limited overall impact on juror's verdict selections following group deliberation. Finally, heightened self‐esteem was found to be significantly associated with guilty verdict selections post‐ (though not pre‐) deliberation. Given that the only known previous study to assess self‐esteem as a possible variable of interest in rape trial juror decision making obtained directly opposing findings (e.g., Willmott 2018), this finding is difficult to reliably determine and also therefore warrants further exploration, especially given that self‐esteem only becomes significant post‐deliberation. Therefore, unlike the role of rape myth beliefs, the current study findings tend to suggest that juror traits including self‐esteem and interpersonal manipulation as well as other variables which were found to have no significant impact upon juror's verdict selections (i.e., parental status, ethnicity, education and victimisation experiences), appear to pose limited prejudicial threat to rape trial jury decision‐making.

### 4.1 Limitations and Strengths

The main limitations of the study relate to the sample and sampling method employed. Due to legal restrictions that largely prohibit research among real jurors in E&W, we cannot confirm whether the use of opportunistically recruited self‐selecting mock jurors fully represent the views of genuine trial jurors. As Thomas (2020) rightly points out, opinion polls suggest that most of the British public do not want to serve as jurors and thus the self‐selecting nature of our participants here *may* distinguish that from genuine jury pools in some latent way. Likewise, as participants recruited for this study were from one Northern English town and were comprised of the general public and university students, the current findings may not be generalisable to the wider UK population. Future research should seek to replicate this study using the same trial materials and survey instruments to examine whether the findings obtained replicate among other samples. Finally, the fact that jurors were aware that the outcome of their decision would not lead to a real defendant being acquitted or convicted differentiated our participants from real jurors. Despite these study limits, the methodology employed in this research represents a substantive improvement compared to much previous research in accordance with several of Willmott et al. (2021) minimum mock trial re‐enactment recommendations. Moreover, we collaborated with various legal professionals to formulate the case and in developed a video‐recorded trial simulation of a genuine rape allegation (as opposed to typically brief written trial vignettes). We also included jury group deliberation and examined the impact this had on juror individual verdict decision making, which almost all previous mock jury research has thus far overlooked.

### 4.2 Implications and Applications

This research has generated important new insights in respect of the impact of juror's rape myth beliefs and the prejudicial impact that they appear to have upon rape trial decision‐making. Indeed, our results indicate that rape myths continue to serve as barriers to rape trial convictions even in less typical cases where the nature of prior acquaintance is disputed. To be clear, even in a case which is arguably weighted in favour of conviction based on the evidence and testimony available, jurors high in rape myth beliefs choose to believe the defendant over the complainant. Our findings subsequently add further weight to on‐going calls for reform to jury trial justice which seek to reduce the influence of rape mythology such as educational interventions (see Hudspith et al. 2024). Despite initial plans in Scotland for juryless trials to be piloted as set out in the ‘Victims, Witnesses, and Justice Reform (Scotland) Bill’ (Scottish Parliament 2023), due to political pressure and threats of strike from the Scottish legal community (Learmonth 2023; Waiton 2023), the proposal was abandoned at the end of 2024 (Cowan and Geddes 2024). Nonetheless, with the Law Commission in E&W (2025) standing firm in their assertion that rape myths do negatively influence jury decision making (a position which sits in stark contrast to claims made by Thomas 2020), further research, and crucially mock trial research that is high in ecological validity (e.g., Weare et al. 2025; Willmott, Boduszek, et al. 2026) is needed to allow for meaningful legal reforms to gain momentum. These study findings make a small contribution to this ever‐developing body of empirical evidence, which over time, may result in jury reforms that afford rape trial complainants and defendants the opportunity for a fair trial where rape myth jury bias is not the key determiner of case outcomes.

## 5 Conclusion

The present study explored the impact of juror characteristics and preexisting beliefs on jury decision‐making in a unique rape case where prior acquaintance is contested. This was investigated through an improved methodological framework exhibiting increased ecological validity. Analyses revealed how in the context of this trial, juror traits including gender, interpersonal manipulation, self‐esteem, and rape myth beliefs (though not ethnicity, parenthood, educational attainment or prior sexual victimisation experience) were differentially with juror decision making at various decision points. However, crucially, in alignment with a wealth of prior research, only jurors rape myth beliefs remained significant predictors of not guilty verdict preferences before and after group deliberation. This further reveals how pre‐trial rape attitudes may serve as prejudicial factors which influence rape trial jury decision making and thus contributes to on‐going calls to reform jury trial justice within rape cases.

## Author Contributions

Both authors (D.W. and R.W.) contributed to all stages of the research including: (1) conception, design, analysis and interpretation of data, (2) drafting of the manuscript, (3) final approval of the work, (4) accountability for the work.

## Funding

The authors have nothing to report.

## Ethics Statement

Ethical approval for the study was obtained from the research ethics committee at the host institution and all study procedures adhered with ethics policies as outlined in the British Psychological Society's (2021) code of human research ethics.

## Conflicts of Interest

The authors declare no conflicts of interest.

## Data Availability

Data is available on request from the lead author.
